# Coddling Children

**Published:** 1900-08

**Authors:** 


### Coddling Children.

Why is it that the people who are most exposed to cold by outdoor employment are the people who are least subject to colds? Simply because no one catches cold by exposing the whole body to cold. A person catches cold when a portion of the body is exposed to cold, while the other portion is kept warm by artificial heat. One is more apt to catch cold sitting by a stove in an unventilated room than by facing a blizzard in the open air.

Nansen and his men, when in the arctic regions, were exposed to cold of every description, and it is stated that they never once suffered from colds. But no sooner had they returned to their native land than they one and all caught severe colds. The reason for this is probably because they were again warmly housed, and spent a portion of their time in unventilated rooms, sleeping in stuffy bedrooms.

The more children are coddled to keep them from catching cold the more apt they are to catch cold. The proper course to take is to clothe the children warmly, provide good, stout shoes, and turn them loose in the open air. Let them go, rain or shine, cold or warm; let them have the open air every day. Such children are far less liable to catch cold. And their bedroom window should be open every night, winter and summer, in such a way as to avoid a direct draft upon them while they are sleeping.—*Medical Talk.*

